# Leadership styles and the process of organizational change during the pandemic

**DOI:** 10.3389/fpsyg.2022.920495

**Published:** 2022-09-12

**Authors:** Atif Saleem, Philip Saagyum Dare, Guoyuan Sang

**Affiliations:** ^1^College of Teacher Education, Zhejiang Normal University, Jinhua, China; ^2^Faculty of Education, Monash University, Melbourne, VIC, Australia; ^3^Center for Teacher Education Research, Beijing Normal University, Beijing, China

**Keywords:** leadership, school principals, pandemic, organizational change, COVID-19

## Introduction

Principals are accustomed to resolving minor crises, confrontations, daily annoyances, and frustrations involving kids, parents, and their own staff. Nonetheless, the COVID-19 epidemic is unique, and the majority of school principals lack experience managing a lengthy and complicated situation (Varela and Fedynich, 2020). Following the proclamation of the SARS-CoV-2 epidemic a pandemic in March 2020 by WHO, normal educational scheduling, activities, and procedures were unexpectedly disrupted and substituted with incertitude. Schools were required to develop new measures to minimize the spread of the infections and safeguard the safety of children and employees (Bailey and Breslin, 2021).

As schools shuttered, countries made a swift shift to online learning (Taglietti et al., 2021). Not only is the school critical for knowledge acquisition and general education, but it is also an integral aspect of the contemporary world (O'Connell and Clarke, 2020). The school system was critical in maintaining the safety and providing care for pupils and their families during the pandemic. Nonetheless, there existed few standardized operational protocols for managing schools in the event of a pandemic and principals were forced to innovate and navigate their schools' activities with or without assistance. Prior leadership research during educational crises has mostly concentrated on how principals respond to severe crises, events, school violence (Pepper et al., 2010).

The global crisis reshaped educational activities, emphasizing more flexible and distributed leadership attributes founded on consensual trust in order to promote both independent and cooperative resilience (Fernandez and Shaw, 2020). Numerous school administrators have been left to juggle the demands of responsible guidance, rapid decision-making, and the need to remain watchful in an unstable circumstances (Netolicky, 2020).

The school institution was woefully unprepared to deal with the pandemic's disruptive impacts. There is a high chance that during a prolonged crisis, the long-term pressure will surpass individual capabilities and available employment resources (Bakker and Demerouti, 2017). Additionally, during the pandemic, the possibility of excessive roles and family-related work conflicts as a result of work-from-home surfaced as a burnout-risk-factor and weariness (Kniffin et al., 2021). Regardless, there is a conjecture that principals are human resource managers and mentors (Wicher, 2017), are obliged to comprehend stakeholders' expectations in varying situations (Brauckmann et al., 2020), and endeavor to meet the ever-increasing and changing needs of students and the community (Gumus et al., 2018), suggesting society looks up to principals during this pandemic.

These expectations around school leaders' resourcefulness during the crisis have resulted in inconsistencies in sustaining a counterbalance of principals' roles that may hamper the optimal school functionality (Huber, 2004), thus, prompted empirical investigations and evidence on crisis of novice (Pineda-Báez et al., 2019), public schools (Mansor et al., 2020), female school leaders (Cruz-González et al., 2020), as well as diversifying stakeholders' expectations (Wong and Liu, 2018). However, none of these empirical evidences suggest path ways for organizations in times of pandemics, there is little or no evidence on the principals' challenges in times of pandemic and how they coped with the changes. Therefore, our review is centered on the rationale that there exists a considerable gap on how principals respond to the process of organizational change (Tamadoni et al., 2021) in pandemic times and in different contexts (Tintoré et al., 2020).

For many firms, organizational transformation has been the norm other than the exemption (Kieselbach et al., 2009). Alterations have been linked to the tendency to discontinue (Oreg, 2006; Holt et al., 2007), decrease productivity, and higher healthcare costs (Mack et al., 1998) and absenteeism (Martin et al., 2005). Instances of change is established to have shown an effect on time constraint, psycho-social wellbeing of followers (Probst, 2003), satisfaction at work (Amiot et al., 2006; Holt et al., 2007), and individual stress (Axtell et al., 2002). However, the pandemic necessitated organizational changes in schools as institutions among all odds where such organizational transformation can bring forth a number of potential consequences (Holten and Brenner, 2015).

With the frequency and breadth of institutional change increasing due to the pandemic, it becomes prudent to explore processes that may lead to good responses to change. While much of the literature on change focuses on the impacts of change, our review focuses completely on how school principals embrace the processes of change. Understanding these pathways will have implications for practice and research. By concentrating only on the process of change; the link between ascendants and denotative reactions, we reply to Semmer (2006) need for assessments of change-intervention-processes since few empirical research have examined favorable receptive reactions to institutional alterations, with the bulk concentrating on the areas where change fails (Oreg et al., 2011). We review the links between transactional and transformational leadership styles as well as change appraisals among followers. In this regard, the present study mainly seeks to address how should principals responded to the institutional change process during the pandemic. To comprehensively understand this concept of change appraisal, the following specific research questions addressed:

- What leadership styles should principals adopt in response to the change process?
- What change mechanisms could guide principals to navigate institutional change?

## Research framework

To comprehensively review and address the change process among teachers and institutional organizations during the pandemic, we adopted Oreg et al. (2011, p. 464) Change Recipient Reactions model which comprises four aspects: the antecedents of pre-change (change characteristics of the recipient, interior context), antecedents of change (process of change, anticipated consequence, change content), explicit responses (behavioral, cognitive, and affective reactions), and lastly, the consequences of change (personal and work-relative). While most studies investigated the negative consequences of change (Oreg et al., 2011), our review focused on antecedent-pathways toward teachers' positive change appraisal development.

**Figure 1:**
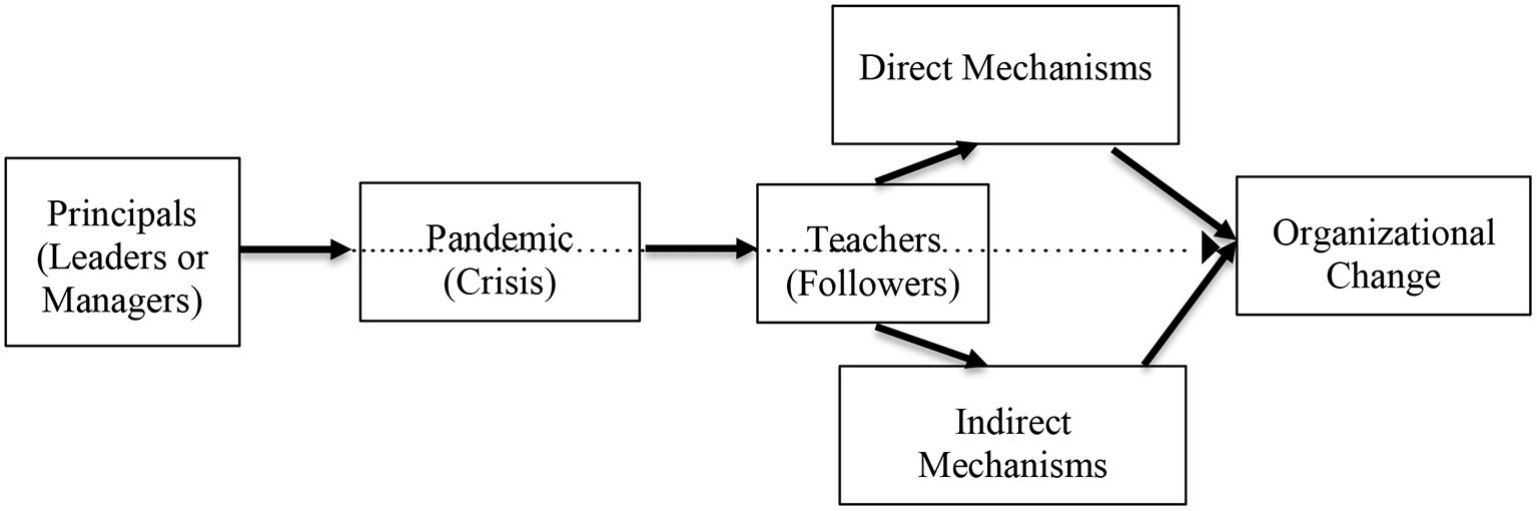
Research framework derived from Holten and Brenner (2015).

With regards to positive teachers' change appraisal development in the process of organizational change, the review strictly followed the suggestions of Holten and Brenner (2015) that leadership styles and change appraisal among followers is directly and indirectly affected by leaders' engagement (derived from the brother framework of Oreg et al., 2011). On the basis of Holten and Brenner (2015), our review projects that the process of organizational change is reinforced by the principals' engagement changes and teachers' direct and indirect change appraisal mechanisms in an attempt to develop positive change appraisal (see Figure 1).

## Positive appraisal change development

Many studies have focused on the antecedents of the change recipient reactions model, which include affective, cognitive, and change readiness expectations, as well as change consequences; job and satisfaction, tendency to quit, and health issues, and depression for instance (Grunberg et al., 2008; Oreg et al., 2011). There are neither pre-change antecedents or post-change consequences addressed in this study, but rather the change intermediate phases, such as the explicit reactions arising from change and the antecedents to change. We reviewed the attitudes and reactions of followers to change (Oreg, 2006; Parish et al., 2008) from the lens of change process. The importance of leadership style and leaders' engagement in fostering favorable views of change is something we reviewed in this study, which builds on previous work. In order to do this, we use the concept of a follower's evaluation of change to investigate both cognitive and behavioral changes. We believe that rather than focusing on the negative effects of change, it is important to keep an eye on people's attitudes about it, how they view the manager leading the change, and how they behave in relation to their daily routines and working methods and traditions.

This review focuses solely on the evaluations and perceptions of teachers as followers. Rather from simply being recipients of change, followers play an active role in influencing it and its effects, therefore, a positive change assessment by followers is thus seen as a necessity for a succeeding in a change process and sustaining positive consequences of change in persons and organizations (Herold et al., 2008; Crawford et al., 2022).

## Leadership styles and engagement changes

Four components define a transformational leadership style: idealized influence, inspiring motivation, independent concern, and intellectual stimulation (Bass, 1985). Transformational leaders serve such as teachers role-model students, foster cooperative goals, encourage confidence, and faith among followers, and inspire them with their leadership style as well as motivate them by encouraging followers to reflect on their traditional practices and beliefs, and provide personal orientations and recognition of personal needs (Bass, 1999).

Two concepts are attributed to transactional leadership style: dependent management and compensation exemption (Bass, 1985) where management by exception factor is subdivided into passive and active management (Lowe et al., 1996). The transactional leadership feature is characterized by an exchange connection between leader and followers where corrective measures are rare and followers get reinforced with rewards for fulfilling certain objectives. Whereas transformational leadership focuses on ideals and visionary leadership, transactional leadership focuses on acknowledging and crediting individual follower-successes.

Transformational vs. transactional leadership literature in most cases examines gender and organizational type patterns: while research on gender dyads indicates that female leaders become more transformative (Bass, 1999; Lien et al., 2022), it also indicates that female followers under female leaders reported higher usage of transformational leadership as compared to their male competitors (Ayman et al., 2009). Lowe et al. (1996) discovered that contrary to their assumptions, transformational leadership and management-by-exception (a type of transactional leadership) are more frequently recorded in public enterprises. The researchers contended if these findings replicate differences in the transformational leadership adopted, the functional requirements, or assessment standards of operation within organizations in the private sector are essential.

Management behavior has an effect on followers' well-being (Skakon et al., 2010), an effect that is amplified during institutional change, during which leaders serve as role models and drivers of change (Kieselbach et al., 2009). Positive responses to change have been demonstrated in organizational change research when administrators become resilient to change, takes a participatory, informed measures, and is viewed as accepted (Oreg et al., 2011). Thus, school principals as managers play a critical role in school transformation, promoting its effectiveness and determining the level at which followers accept non-traditional situations (Armenakis et al., 2007).

We especially present the influence of leadership and changes involving the process through which followers (teachers) generate such favorable assessments of change. Transformational leadership is noted as an effective type of leadership that appreciate navigating organizational transformation (Eisenbach et al., 1999; Lien et al., 2022). The leadership approach enables followers to contain non-conventional situations more effectively (Callan, 1993) and strengthens commitment of followers and empower self-efficacy in times of transition (Bommer et al., 2005).

The transformational and transactional approaches to leadership are distinct but compatible: the transformational approach, in terms of augmenting effect of ideas, provides the foundation for and enhances the effects derived from the transactional approach to leadership (Avolio, 1999; Lien et al., 2022). In transitional times, charismatic (transformational) approach to leadership serves as a mental anchor for followers by role-modeling who demonstrates acceptable behaviors.

Instrumental (transactional) approach to leadership assures the commitment produced by charismatic leadership behavior, adhered to and maintained (Nadler and Tushman, 1990). We enhance existing pragmatic research by extending the notion hence establishing trend of literature on principals (managers engagement).

## Indirect factors underlying followers' change appraisal

To ensure change is effective, leaders should strive to connect their stated and implemented principles (Eisenbach et al., 1999). Such alignment is referred to as behavioral integrity (Simons, 2002). Within the framework of integrity theory on behavior, transformational and transactional approaches to leadership are viewed as the stated values of managers, whereas change engagement is viewed as the practiced values of managers. Thus, both leadership approaches within the frame of the professed values, would match with distinguished change engagement of particular managers within the structure of enacted values in effective change processes.

During times of organizational transition, like in the pandemic instance, transformational and transactional leadership styles complement each other (Nadler and Tushman, 1990). By Simons (2002), transformational leadership enables successful alteration through fostering trustworthiness and credibility, which are fostered by integrity of behavior. As a result, we argue that transformational approach to leadership will significantly impact change-oriented engagement of managers. Transactional approach to leadership is instrumental and provides a tangible fountain where leaders may actively involve followers in achieving the desired change. Transactional leadership self-empowering and crediting attribute underlies some engagement attitudes including information dissemination and defining personal impact.

Much study on leadership behaviors associated with organizational transformation has concentrated on the acceptance and commitment of followers. Herold et al. (2008) discovered a positive correlation between change management and followers' commitment to change, whereas Aarons (2006) discovered that receptivity may increase in situations where there is a local opinion leader who is viewed positively, initiate and foster change conditions. The study of Kavanagh and Ashkanasy (2006) discovered that acceptance and or objection to change among followers was affected by the change management technique.

By examining the evolution of followers' change evaluation, we improve past studies. Developing a positive change assessment system would be a critical indication of tendencies of effectiveness of processes of change and, consequently, of good human and cooperative results. Thus, the methods preceding this metric are critical and interesting for companies that are planning and executing change.

## Direct mechanisms underlying followers' evaluation of change

Additionally, this article reviewed how leadership directly affect the perceptions of change among followers. While transformational approach to leadership is associated with effective change implementation (Oreg et al., 2011), the transactional approach is appropriate in situations when the status quo is maintained while particular goals are achieved (Gersick, 1994). According to Eisenbach et al. (1999), the transformational type of leadership is appropriate for organizational change and possesses favorable impact on the reactions of followers to institutional changes (Oreg et al., 2011).

The process of transformational leadership motivates followers to alter their attitudes and assumptions while fostering devotion to corporate goals (Yukl, 1989). Studies of Holten and Brenner (2015) claimed that transformational approach to leadership correlates with maximizing the process of change, as certified by the modification of schedules and methods, the elimination of inefficient work practices, and the modification of attitudes toward the team and its ability to manage institutional changes. Thus, we suggest that transformational approach to leadership will result in a significant shift in the perceptions of followers toward change.

While the transformational type is associated with a favorable assessment of change among followers, the transactional type, which is largely motivated by extrinsic motivation, encourages compliance of followers in duties via bonuses and inducements (Bass, 1985).

Through reinforcement and incentive, transactional approach to leadership is presumed to motivate change acceptance throughout organizational transition. Such instances, however, would be a means to an end rather than attitude-oriented. Whereas the transactional type of leadership is appropriate for businesses that prefer to retain the situation (Gersick, 1994), it is not expected of the transactional approach to have a favorable effect on followers' perceptions of change. In situations where the transactional leadership implies a failing to drive followers across the anticipated goals, it would appear impossible to influence a positive evaluation during times of uncertainty.

## Discussion

Our review was centered on the leadership styles of school principals in the process of organizational change during the pandemic. The review suggested that the process of organizational change is reinforced by followers' development of positive appraisal about change, including both indirect and direct mechanisms to followers' change appraisal as leadership styles and leaders' engagement during the change is eminent. The studies of Holten and Brenner (2015), Edelbroek et al. (2019), and Azizaha et al. (2020) suggest both transactional and transformational leadership styles positively correlate with the change engagement of leaders during the process of organizational change implying. This implies, the success of organizational change among teachers during the pandemic depends on the leadership styles of their respective schools – thus, if their principals employed either transactional or transformational types of leadership, they were more likely to smoothly transition through organizational change.

In congruence with Oreg et al. (2011) and Herrmann et al. (2012), organizational change is considered a consequence of transformational leadership as it assumed to reinforce leadership and follower attitudes through active and conservative urge for change in the change process as suggested by Herscovitch and Meyer (2002). Evidence of such characteristics pushes both leaders and followers to perform beyond expectations in view of the Bass (1999) conceptualization during the change process although Vakola and Nikolaou (2005) indicated it can be psychologically difficult to adapt. On basis of transformational leadership, both principals and teachers are deemed to perform above expectations in attempts to embrace change.

In addition, the review reported the process of organizational change among teachers were determinable by both indirect and direct mechanisms to appraisal of change; commitment, and receptivity of followers (Aarons, 2006; Herold et al., 2008), rewards and incentives (Saqib et al., 2015; Khan et al., 2018). Implacably, for school principals and teachers to undergo the sudden change as a consequence of the pandemic, the teachers as followers should be willing to be committing and reaccepting which indirectly reinforce the success of the change process.

On the other hand, school principals can directly influence the change process by using rewards and incentives to motivate teachers to be committed and receptive in the change process. It can be concluded organizational change can be attained through transactional and transformational leadership as well as the use of rewards and incentives. In view of Faupel and Süß (2019), followers perceptive captivating change consequences in the transformational leadership paradigm motivates them (followers and employees) to actively embrace and foster change through leadership-follower behavior. That is, followers' tendency to perceive that there is significant outcome in a change process, Faupel and Süß (2019), indicated they (followers) are more likely to behave in the direction of change.

## Conclusion

In sum, the experiences of school principals during a prolonged crisis demonstrate that effective situational management requires a variety of managerial duties, including honest communication, dispersed leadership, acquiring knowledge from uncertainties, and making of decisions ambiguously. In an eventual scenario comprising the pandemic, principals of school were forced to adjust their leadership approaches to meet extraneous obligations as well as the teachers and pupils' internal needs. As a result, the roles of the school principals grew more complicated. School principals became managers of their institutions and acted out managerial roles. The loneliness of command became apparent during the crises, highlighting the need of crisis planning and the ways in which school organizations and authorities might learn and grow from the pandemic experience. To respond to such crises, principals adopted transformational and transactional leadership strategies alongside both direct and indirect change mechanisms to foster.

However, as an opinion paper, the discursive review is the researchers' viewpoint of analytical literature and might not applicable in empirical contexts. In light of this, we therefore recommend that future studies should adopt empirical methods and quantitative approaches to measure the level of effect that transactional and transformational leadership styles exert on organizational change. Such studies can also examine the impact of incentives and rewards on organizational change when used in either transactional or transformational leadership.

## Author contributions

All authors listed have made a substantial, direct, and intellectual contribution to the work and approved it for publication.

## Funding

This study was funded by the International Joint Research Project of Huiyan International College, Faculty of Education, Beijing Normal University (ICER202001).

## Conflict of interest

The authors declare that the research was conducted in the absence of any commercial or financial relationships that could be construed as a potential conflict of interest.

## Publisher's note

All claims expressed in this article are solely those of the authors and do not necessarily represent those of their affiliated organizations, or those of the publisher, the editors and the reviewers. Any product that may be evaluated in this article, or claim that may be made by its manufacturer, is not guaranteed or endorsed by the publisher.
